# From Defense Executor to Engineering Target: Harnessing Lignin for Crop Resistance

**DOI:** 10.1002/advs.76059

**Published:** 2026-06-12

**Authors:** Yanwen Yu, Ke Wang, Huachuan Tuo, Jiankun Li, Liping Xu, Chang‐Jun Liu, Mingyue Gou

**Affiliations:** ^1^ State Key Laboratory of High‐Efficiency Production of Wheat‐Maize Double Cropping Collaborative Innovation Center of Henan Grain Crops Center for Crop Genome Engineering College of Agronomy Henan Agricultural University Zhengzhou China; ^2^ Biology Department Brookhaven National Laboratory Upton New York USA; ^3^ The Shennong Laboratory Zhengzhou Henan China

**Keywords:** crop engineering, defense executor, lignin, pathogen and pest resistance, regulatory mechanisms

## Abstract

Plants have evolved sophisticated defense mechanisms involving pathogen and pest perception, signal transduction, and immunity execution. While immune receptors and signaling components underlying the first two steps have been extensively studied over the past three decades, the downstream physical and chemical executors that plants deploy to combat pathogen invasion have received comparatively less attention. Lignin is a fundamental structural polymer in the plant secondary cell wall. In recent years, an increasing number of studies have demonstrated that plants actively employ lignin fortification as a defense strategy against a broad spectrum of biotic stresses, underscoring lignin's crucial role in immunity execution. In this review, we comprehensively summarize the genetic contributions of lignin‐related genes to plant immunity and elucidate the regulatory mechanisms governing the dynamic activation of monolignol biosynthesis, transport, and polymerization following pathogen/pest attack. In addition, we discuss the physiological and biochemical basis of lignin‐mediated immunity and highlight outstanding questions in the field. Furthermore, we summarize recent advances in enhancing crop resistance through biotechnological modulation of lignin content and composition. Overall, this review underscores the role of lignin as a stalwart defense executor and positions the lignin pathway as a strategic target for crop engineering toward enhanced resistance against diverse biotic threats.

## Graphical abstract

Lignin serves as both a physical and chemical executor of broad‐spectrum plant immunity. This review systematically summarizes classical and recent advances in lignin‐mediated immunity, outlines the multifaceted regulatory modules controlling lignification under biotic stress, discusses key physiological and biochemical mechanisms behind lignin‐based defense, and proposes a practical framework for modulating lignin to improve crop resistance.

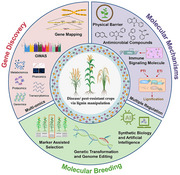

## 1 Introduction

Pathogens and pests are critical factors that limit plant growth and production, significantly reducing crop yield and posing a serious threat to global food security. According to the Food and Agriculture Organization (FAO), annual global food production is reduced by up to 40% due to these challenges [1]. Therefore, breeding crop varieties with enhanced resistance to biotic stresses is crucial for sustainable agriculture. To defend themselves against diverse biotic stresses, plants have evolved a three‐step immune response system that includes pathogen and pest perception by upstream immune receptors, signal transduction mediated by intermediate signaling components, and the immunity execution via downstream physical and chemical executors [2]. Over the past three decades, pathogen perception and immune signaling have been extensively studied. These efforts have revealed the roles of cell‐surface pattern recognition receptors (PRRs) in pattern‐triggered immunity (PTI) and intracellular nucleotide‐binding leucine‐rich repeat receptors (NLRs) in effector‐triggered immunity (ETI), as summarized in several reviews [3, 4, 5, 6]. Activation of both PTI and ETI leads to diverse physical and chemical actions that directly combat pathogen invasions. However, these downstream executors of plant immunity have received comparatively less attention.

Plant cell walls are complex and dynamic structures composed primarily of high molecular weight polysaccharides, highly glycosylated proteins, and lignin [7]. Lignin is an aromatic polymer that interacts with cellulose and hemicellulose to form an extracellular matrix in the secondary cell walls of specific tissues, including the periderm, phloem fibers, sclerenchyma, and xylem, imparting rigidity and impermeability [8]. As the second most abundant biopolymer in plants, lignin is unique to vascular plants and accounts for approximately 30% of the organic carbon content in the terrestrial biosphere [9]. Lignin plays pivotal roles in plant growth and development by enhancing the rigidity and hydrophobicity of cell walls, providing structural support, and enabling long‐distance transport of water and nutrients [8, 10, 11]. Additionally, lignin‐enriched cell walls are highly resistant to physical disruption and enzymatic degradation by microbes and pests, while drought, salinity, and cold can also induce dynamic changes in lignin content and composition. Lignification is thus a key defense mechanism against various biotic and abiotic stresses [12, 13, 14].

In particular, lignin has long been recognized as a physical barrier against pathogens and pests [12, 15, 16]. Over the past decades, lignin has attracted increasing attention, with significant advances in the modification of its biosynthetic pathway in various plants [13, 14, 17]. Notably, in the recent two years, there has been a surge of publications highlighting lignin's key role in plant defense, particularly in broad‐spectrum disease resistance in major crops such as rice, wheat, and maize. These studies have revealed sophisticated regulatory mechanisms governing lignin‐mediated immunity throughout the entire lignification process [18, 19, 20, 21, 22, 23, 24, 25, 26, 27, 28, 29, 30, 31], emphasizing lignin as a robust defense executor and a key engineering target for crop resistance improvement. However, there is a lack of an integrative review of the old theory and new findings, especially on the multi‐layered regulatory mechanisms of lignin‐mediated immunity and the application in crop resistance improvement. Here, we systematically summarize the classical understanding and recent advances in lignin‐mediated plant immunity, which involves 73 genes conferring resistance to 43 pathogens and pests in 20 plant species (Table 1). We also distill regulatory modules controlling lignin biosynthesis, transport, and polymerization in response to pathogen/pest attacks and propose a conceptual framework for enhancing plant resistance through genetic manipulation of lignin‐related genes. This framework aims to facilitate molecular breeding of crops with coordinated improvements in both disease resistance and yield, thereby bridging the gap between fundamental research and practical applications in crop improvement.

**TABLE 1:** Disease and Pest Resistance of Plants with Altered Lignin Content and Composition.

Gene	Plant species	Pathogen/Pest Tested	Category	Mutant/Transgene	Lignin	Resistance	Reference
**Monolignol Biosynthetic Genes**							
*PAL*	Arabidopsis	*Pst*	Hemibiotrophic bacteria	*palQ*	Decrease	S	[69, 104]
	Tobacco	*Phytophthora parasitica*; *Cercospora nicotianae*	Hemibiotrophic fungi Necrotrophic fungi	OE‐*ShPAL*	Increase	R	[106]
		Tobacco mosaic virus	Virus	OE‐*PAL2*	Increase	R	[105]
	Rice	*Magnaporthe oryzae*	Hemibiotrophic fungi	OE‐*OsPAL1*	Increase	R	[107, 108]
		*Nilaparvata lugens*; *Xoo*; *Magnaporthe oryzae*	Pest Biotrophic bacteria Hemibiotrophic fungi	OE‐*OsPAL6*	Increase	R	[21]
*HCT*	Arabidopsis			RNAi‐*HCT*	Decrease	ADR	[112]
	Alfalfa	*Colletotrichum trifolli*	Hemibiotrophic fungi	RNAi‐*HCT*	Decrease	R	[109]
*CCR*	Arabidopsis	*Pst*	Hemibiotrophic bacteria	*ccr1‐6*	Decrease	S	[68]
				*ccr1‐3*	Decrease	ADR	[112]
	*Brassica napus*	*Sclerotinia sclerotiorum*	Necrotrophic fungi	OE‐*BnCCR2*	Increase	R	[113]
*4CL*	Cotton	*Verticillium dahliae*	Hemibiotrophic fungi	VIGS‐*4CL30*	Decrease	R	[116]
		*Verticillium dahliae*	Hemibiotrophic fungi	CR‐*Gh4CL3*	Decrease	S	[115]
				OE‐*Gh4CL3*	Increase	R	
	Chrysanthemum	*Macrosiphoniella sanborni*	Pest	RNAi‐*Cm4CL2*	Increase	R	[61]
				OE‐*Cm4CL2*	Decrease	S	
*CCoAOMT*	Arabidopsis	*Pst*; *Hyaloperonospora arabidopsidis*	Hemibiotrophic bacteria Biotrophic fungi	*ccoaomt1‐3*/5	Decrease	S	[117]
	Maize	*Bipolaris maydis*	Necrotrophic fungi	OE‐*ZmCCoAOMT2*	Increase	R	[117]
*COMT*	Arabidopsis	*Hyaloperonospora arabidopsidis*	Biotrophic fungi	*comt1*	Decrease	R	[120]
		*Botrytis cinerea*; *Alternaria brassicicola;* *Xanthomonas campestris;* *Pst*	Necrotrophic fungi Necrotrophic fungi Hemibiotrophic bacteria Hemibiotrophic bacteria	*comt1*	Decrease	S	[120]
	Sorghum	*Fusarium* spp.	Hemibiotrophic fungi	*bmr12*	Decrease	R	[119]
	Maize	*Ustilago maydis;* *Setosphaeria turcica;* *Pantoea stewartii;* *Colletotrichum graminicola;* *Fusarium graminearum;* *Cercospora zeae‐maydis*	Biotrophic fungi Hemibiotrophic fungi Biotrophic fungi Hemibiotrophic fungi Hemibiotrophic fungi Necrotrophic fungi	*bm3*	Decrease	S	[121, 122]
	Wheat	*Puccinia striiformis*	Biotrophic fungi	VIGS*‐TaCOMT‐3B*	Decrease	S	[27]
	Tobacco	*Agrobacterium tumefaciens*	Biotrophic bacteria	RNAi‐*COMT*	Decrease	R	[118]
*F5H*	Arabidopsis	*Sclerotinia sclerotiorum*	Necrotrophic fungi	*fah1‐2*	G/S increase	S	[123]
		*Verticillium longisporum*	Necrotrophic fungi	*fah1‐2*	G/S increase	S	[124]
		*Pst*	Hemibiotrophic bacteria	OE‐*F5H*	G/S decrease	R	[63]
		*Myzus persicae*	Pest	OE‐*F5H*	G/S decrease	S	
*CAD*	Arabidopsis	*Pst*	Hemibiotrophic bacteria	*cad‐C cad‐D*	Decrease	S	[174]
	Tobacco	*Trichobaris mucorea*	Pest	RNAi‐*CAD*	Decrease	S	[51]
	Flax	*Fusarium oxysporum*	Hemibiotrophic fungi	RNAi‐*CAD*	Decrease	S	[125]
	Persimmon	*Colletotrichum horii*	Hemibiotrophic fungi	OE‐*DkCAD1*	Increase	R	[89]
	Rose	*Botrytis cinerea*	Necrotrophic fungi	OE‐*RhCAD1*	Increase	R	[128]
	Sorghum	*Fusarium* spp.	Hemibiotrophic fungi	*bmr6*	Decrease	R	[175]
	Rice	*Rhizoctonia solani*	Necrotrophic fungi	CR‐*OsCAD8B*	Decrease	S	[126]
		*Magnaporthe oryzae*	Hemibiotrophic fungi	*oscad2*	Decrease	S	[127]
				OE‐*OsCAD2*	Increase	R	
	Wheat	*Bsorokiniana Sorokiniana*; *Fusarium pseudograminearum*	Hemibiotrophic fungi Hemibiotrophic fungi	OE‐*TaCAD1*	Increase	R	[18]
	Maize	*Rhizoctonia solani*	Necrotrophic fungi	*zmcad*	Decrease	S	[126]
		*Ustilago maydis* *Setosphaeria turcica;* *Pantoea stewartii;* *Colletotrichum graminicola;* *Fusarium graminearum;* *Cercospora zeae‐maydis*	Biotrophic fungi Hemibiotrophic fungi Biotrophic fungi Hemibiotrophic fungi Hemibiotrophic fungi Necrotrophic fungi	*bm1*	Decrease	S	[121, 122]
**Monolignol Transport and Polymerization Related Genes**							
*Lr34*	Wheat	*Puccinia striiformis*	Biotrophic fungi	*m19*; *m21*	Decrease	S	[27]
*LAC*	Arabidopsis	*Verticillium dahliae*	Hemibiotrophic fungi	OE‐*GhLAC15*	Increase	R	[74]
	Cotton	*Verticillium dahliae*	Hemibiotrophic fungi	VIGS‐*GhLAC15*	Decrease	S	[74]
		*Verticillium dahliae*	Hemibiotrophic fungi	VIGS‐*GhLAC4*	Decrease	S	[91]
	Kiwifruit	*Psa*	Biotrophic bacteria	OE‐*AcLAC35*	Increase	R	[129]
	*Camellia sinensis*	*Pcs*	Hemibiotrophic fungi	OE‐*CsLAC17*	Increase	R	[94]
CASPL	Arabisopsis	*Psa*	Hemibiotrophic bacteria	*caspl4d1‐1/2*; RNAi‐*CASPL1D*	Decrease	S	[82]
*DIR*	Cotton	*Verticillium dahliae*	Hemibiotrophic fungi	OE‐*GhDIR1*	Increase	R	[76]
	Wheat	*Fusarium pseudograminearum*	Hemibiotrophic fungi	VIGS‐*DIR*; *dir*	Increase	R	[77]
**Lignin Biosynthesis Regulatory Genes**							
*MYB*	Arabidopsis	*Pst*	Hemibiotrophic bacteria	*myb15‐1/2;*	Decrease	S	[82]
		*Xoo*; *Xcc*	Biotrophic bacteria Hemibiotrophic bacteria	OE‐*MYB4* CR‐*MYB4*	Decrease Increase	S R	[101]
	Rice	*Xoo*; *Xcc*	Biotrophic bacteria Hemibiotrophic bacteria	CR‐*MYB102*/108	Increase	R	[101]
				OE‐*MYB102*	Decrease	S	
		*Magnaporthe oryzae;* *Rhizoctonia solani;* *Xoo*	Hemibiotrophic fungi Necrotrophic fungi Biotrophic bacteria	OE‐*MYB30* *myb30‐1/2*	Increase Decrease	R S	[24]
	Maize	*Fusarium graminearum*	Hemibiotrophic fungi	*zmmyb31*	Increase	R	[131]
		*Fusarium graminearum;* *Pythium inflatum*	Hemibiotrophic fungi Necrotrophic fungi	*RNAi‐ZmMYB74*	Increase	R	[31]
				*OE‐ZmMYB74*	Decrease	S	
	Chrysanthemum	*Macrosiphoniella sanborni*	Pest	RNAi‐*CmMYB15*	Decrease	S	[84]
				OE‐*CmMYB15*	Increase	R	
	Cotton	*Verticillium dahliae*	Hemibiotrophic fungi	VIGS‐*GhODO1*	Decrease	S	[88]
		*Verticillium dahliae*	Hemibiotrophic fungi	OE‐*GhMYB3D5*	Increase	R	[86]
				VIGS‐*GhMYB3D5*	Decrease	S	
		*Verticillium dahliae*	Hemibiotrophic fungi	OE‐*GhMYB4*	Decrease	R	[87]
	Apple	*Colletotrichum gloeosporioides*	Hemibiotrophic fungi	OE‐*MdMYB46*	Increase	R	[20]
				RNAi‐*MdMYB46*	Decrease	S	
*WRKY*	Cotton	*Verticillium dahliae*	Hemibiotrophic fungi	RNAi‐*GhWRKY1L*	Decrease	S	[65]
				OE‐*GhWRKY1L*	Increase	R	
		*Verticillium dahliae*	Hemibiotrophic fungi	VIGS‐*GhWRKY55*	Increase	R	[176]
	Rose	*Botrytis cinerea*	Necrotrophic fungi	VIGS‐*RhWRKY30*	Decrease	S	[128]
	Persimmon	*Colletotrichum horii*	Hemibiotrophic fungi	OE‐*DkWRKY*	Increase	R	[89]
	Rice	*Nilaparvata lugens*; *Xoo*; *Magnaporthe oryzae*	Pest Biotrophic bacteria Hemibiotrophic fungi	CR‐*OsWRKY36* OE‐*OsWRKY36*	Increase Decrease	R S	[21]
SPL	Kiwifruit	*Psa*	Hemibiotrophic bacteria	OE‐*AcSPL9*	Increase	R	[129]
				VIGS‐ *AcSPL9*	Decrease	S	
*SNB*	Rice	*Magnaporthe oryzae;* *Rhizoctonia solani;* *Xoo*	Hemibiotrophic fungi Necrotrophic fungi Biotrophic bacteria	OE‐*SNB* *snb‐1*	Decrease Increase	S R	[24]
*RD26*	Arabidopsis	*Ralstonia solanacearum*	Hemibiotrophic bacterial	*rd26‐1*/2	Decrease	S	[24]
				OE*‐RD26*	Increase	R	
	Tomato	*Ralstonia solanacearum*	Hemibiotrophic bacterial	*RNAi‐SlRD26*	Increase	R	[24]
*HB16*	Cassava	*Xam*	Hemibiotrophic bacterial	OE*‐MeHB16*	Increase	R	[26]
				RNAi*‐MeHB16*	Decrease	S	
*ERF114*	Apple	*Fusarium solani*	Hemibiotrophic fungi	OE‐*MdERF114*	Increase	R	[130]
				RNAi‐*MdERF114*	Decrease	S	
*MKP1*	Arabidopsis	*Xoo;* *Xcc*	Biotrophic bacteria Hemibiotrophic bacteria	*mkp1*; CR*‐MKP1*	Decrease	S	[101]
	Rice	*Xoo;* *Xcc*	Biotrophic bacteria Hemibiotrophic bacteria	OE*‐OsMPK1* CR*‐OsMPK1*	Increase Decrease	R S	[101]
*MPK*	Arabidopsis	*Xoo*	Biotrophic bacteria	*mpk3*; *mpk6*	Increase	R	[101]
*FERONIA*	Arabidopsis	*Ralstonia solanacearum*	Hemibiotrophic bacterial	*fer*	Increase	R	[23]
	Tomato	*Ralstonia solanacearum*	Hemibiotrophic bacterial	RNAi*‐SlFER*	Increase	R	[23]
	Tobacco	*Ralstonia solanacearum*	Hemibiotrophic bacterial	Cr*‐NtFER*	Increase	R	[23]
*PTI1c*	Maize	*Fusarium graminearum*	Hemibiotrophic fungi	*pti1c*	Increase	R	[131]
*miRNA*	Malus hupehensis	*Botryosphaeria dothidea*	Hemibiotrophic fungi	OE‐*MhmiR397b*	Decrease	S	[92]
				STTM‐*miR397*	Increase	R	
	Cotton	*Verticillium dahliae*	Hemibiotrophic fungi	OE‐*miR397*	Decrease	S	[91]
				STTM‐*miR397*	Increase	R	
	*Camellia sinensis*	*Pcs*	Hemibiotrophic fungi	OE‐*CsmiR397a*	Decrease	S	[94]
	Rice	*Magnaporthe oryzae;* *Rhizoctonia solani;* *Xoo*	Hemibiotrophic fungi Necrotrophic fungi Biotrophic bacteria	OE‐*miR172a* *mir172a*	Increase Decrease	R S	[24]
*BSR‐K1*	Rice	*Magnaporthe oryzae;* *Xoo*	Hemibiotrophic fungi Biotrophic bacteria	*bsr‐k1*	Increase	R	[108]
*PUB41*	Rice	*Magnaporthe oryzae*	Hemibiotrophic fungi	CR*‐OsPUB41*	Increase	R	[103]
				OE‐*OsPUB41*	Decrease	S	
*FBK16*	Rice	*Magnaporthe oryzae*	Hemibiotrophic fungi	*osfbk16*	Increase	R	[107]
*FJ10*	Rice	*Nilaparvata lugens*	Pest	*jaz10‐3;* OE‐*FJ10*	Increase	R	[19]
*FBL41*	Maize	*Rhizoctonia solani*	Necrotrophic fungi	*zmfbl41*	Increase	R	[126]

OE, Overexpression; RNAi, RNA interference; CR, CRISPR/Cas9 gene editing; VIGS, Virus‐Induced Gene Silencing; STTM, short tandem target mimic; R, enhanced resistance; S, increased susceptibility; ADR, Activated defense response; *Pst*, *Pseudomonas syringae* pv. *tomato*; *Xoo*, *Xanthomonas oryzae* pv. *oryzae*; *XCC*, *Xanthomonas campestris* pv. *campestris*, *Xam, Xanthomonas axonopodis* pv. *manihotis*; *Psa, Pseudomonas syringae* pv. *actinidiae*; *Pcs*, *Pseudopestalotiopsis camelliae‐sinensis*.

## 2 A Brief Overview of the Lignification Process

**FIGURE 1:**
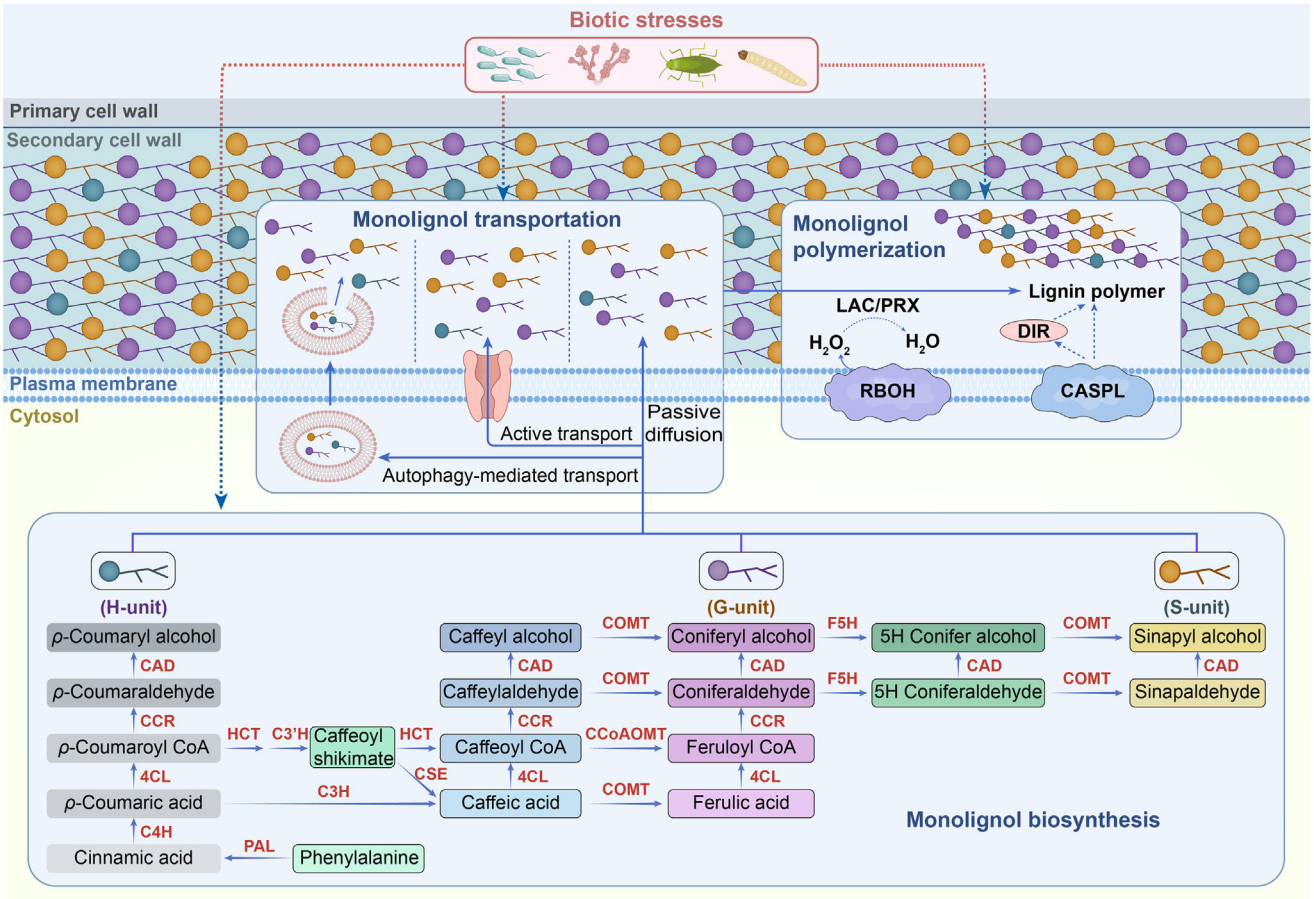
Schematic Representation of the Plant Lignification Process: Monolignol Biosynthesis, Transport, and Polymerization. PAL, phenylalanine ammonia‐lyase, C4H, cinnamic acid 4‐hydroxylase, 4CL, 4‐coumarate‐CoA ligase, HCT, shikimate hydroxycinnamoyl transferase, C3'H, coumaroyl shikimate 3'‐hydroxylase, C3H, coumarate 3‐hydroxylase, CSE, caffeoyl shikimate esterase, F5H, ferulate 5‐hydroxylase, COMT, caffeic acid 3‐*O*‐methyltransferase, CCoAOMT, caffeoyl‐CoA 3‐*O*‐methyltransferase, CCR, cinnamoyl‐CoA reductase, CAD, cinnamyl alcohol dehydrogenase, LAC, O_2_‐dependent laccase, PRX, H_2_O_2_‐dependent peroxidase, RBOH, respiratory burst oxidase homolog, CASPL, Casparian strip membrane domain protein like, DIR, dirigent protein.

Lignification in plant cells comprises three sequential stages: (1) monolignol biosynthesis, (2) transport, and (3) polymerization [32, 33]. (Figure 1)

Monolignols, the fundamental building blocks of lignin polymers, are synthesized in the cytoplasm through the phenylpropanoid pathway, with the three primary units being *ρ*‐coumaryl, coniferyl, and sinapyl alcohols (H, G, and S units) [8, 34]. Biosynthesis begins with the conversion of phenylalanine to *ρ*‐coumaroyl‐CoA by phenylalanine ammonia‐lyase (PAL), cinnamic acid 4‐hydroxylase (C4H), and 4‐coumarate‐CoA ligase (4CL), followed by reactions involving the P450 enzymes *ρ*‐coumaroyl shikimate 3′‐hydroxylase (C3′H) and ferulate 5‐hydroxylase (F5H), the methyltransferases caffeic acid 3‐*O*‐methyltransferase (COMT) and caffeoyl‐CoA 3‐*O*‐methyltransferase (CCoAOMT), and the reductases cinnamoyl‐CoA reductase (CCR) and cinnamyl alcohol dehydrogenase (CAD) to produce monolignols [33, 34]. The three P450 proteins C4H, C3′H, and F5H form protein complexes involving two non‐catalytic membrane steroid‐binding proteins (MSBPs) [35], and they hydroxylate the aromatic ring with electron donors cytochrome P540 reductase (ATR1 and ATR2) and the cytochrome *b*_5_ isoform CB5D [36, 37, 38].

After being synthesized in the cytoplasm, monolignols are transported to the cell wall for subsequent polymerization into lignin polymers. Several hypotheses have been proposed for the mechanism of monolignol transport [39]. Monolignol was originally proposed to be transported by a specific ATP‐binding cassette (ABC) transporter, such as AtABCG29 and Lr34, which function as ρ‐coumaryl alcohol and sinapyl alcohol transporters in Arabidopsis and wheat, respectively [27, 40, 41]. Besides, monolignols may cross the plasma membrane via passive diffusion. Molecular dynamics simulations indicate that several monolignols and related compounds can diffuse through the membrane at rates sufficient to support lignin biosynthesis [42, 43]. Notably, this diffusion is enhanced when a concentration gradient is established by the presence of laccases on one side of the membrane [44]. In addition, the colocalization of pathogen‐induced monolignols and autophagic vesicles suggests that monolignol transport could be mediated by autophagic membrane trafficking, and this autophagy‐mediated transport is an emergency mechanism for plants to rapidly transport induced monolignols as part of their immune response [45].

Finally, polymerization of monolignols after oxidation catalyzed by O_2_‐dependent laccases (LACs) and H_2_O_2_‐dependent peroxidases (PRXs) is achieved via combinatorial radical coupling reactions [46]. The polymerization of lignin in specific domains or structures like the Casparian strip is guided by dirigent proteins (DIRs) and Casparian strip membrane domain proteins (CASPs) [47]. CASPs act as a transmembrane protein scaffold for the localization of lignin‐related enzymes, which oxidize monolignols to generate their corresponding radicals. These radicals are further utilized by DIR heterotrimers to form dimerization products [47]. Recent advances have progressively revealed the complexity of the whole lignification process, with comprehensive reviews providing clear mechanistic insights [33, 34, 39, 46, 48].

## 3 Lignification Acts as an Inducible and Dynamic Defense Strategy Against Pathogens and Pests

**FIGURE 2:**
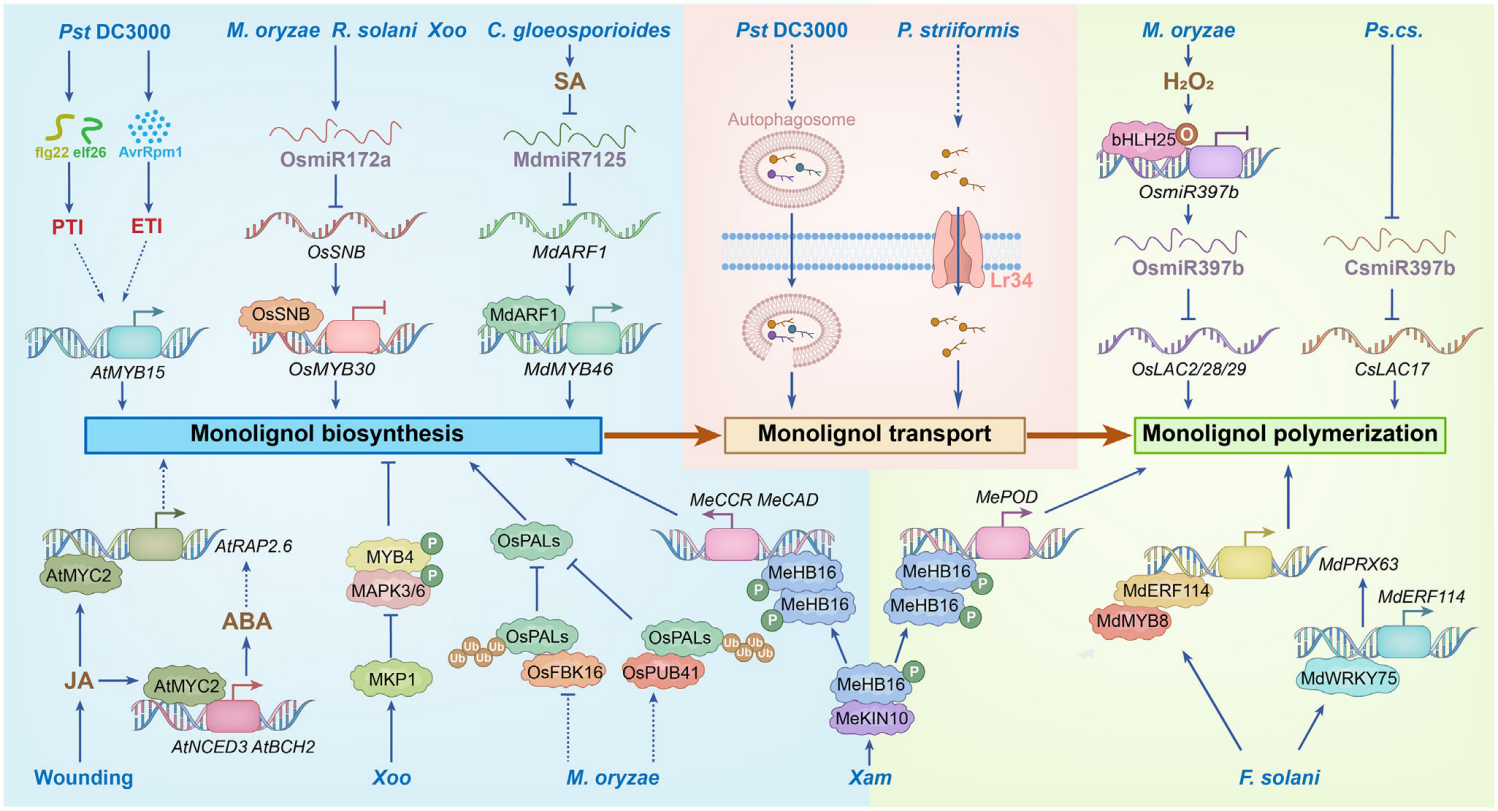
Regulatory Modules Involved in Pathogen/Pest‐activated Lignification. *Pst*, *Pseudomonas syringae* pv. *tomato*, *M. oryzae*, *Magnaporthe oryzae*, *R. solani*, *Rhizoctonia solani*, *Xoo*, *Xanthomonas oryzae* pv*. oryzae*, *C. gloeosporioides*, *P. striiformis*, *Puccinia striiformis*, *Colletotrichum gloeosporioides*, *Ps.cs*., *Pseudopestalotiopsis camelliae‐sinensis*, *Xam*, *Xanthomonas axonopodis* pv. *manihotis*. *F*. *solani*, *Fusarium solani*, PTI, pattern‐triggered immunity, ETI, effector‐triggered immunity, SA, salicylic acid, ABA, abscisic acid, JA, jasmonic acid, P, phosphoryl, Ub, ubiquitin, O, oxidization.

In addition to constitutive lignin deposition, which provides passive defense, plants can dynamically induce lignin accumulation under stress conditions or deposit it in specialized tissues as an active defense mechanism. This inducible lignin is commonly referred to as “stress lignin” or “defense lignin” [49]. Unlike the fixed distribution of structural lignin in supportive tissues such as xylem vessels and fibers, defense lignin is specifically deposited at the infection site. For example, lignin is rapidly synthesized and deposited in papillae‐specialized structures that form around pathogen penetration sites. These lignin‐rich papillae function as a physical barrier that restricts microbial invasion [50]. The polymerization of defense lignin is more rapid and usually has compositional differences compared to structural lignin. For instance, a decreased S/G ratio, increased *β*‐aryl ether (*β–*O–4) lignin units, and increased feruloyltyramine as a newly appearing lignin component are found in the pith of tobacco attacked by stem‐boring herbivores [51]. The differences between defense lignin and structural lignin in spatial patterns, polymerization kinetics, and compositions enable defense lignin to function as a specialized defense executor, distinct from structural lignin, which mainly serves a supportive role. Meanwhile, increasing studies have reported that lignification is promoted by the activation of monolignol biosynthesis, transport, and polymerization upon pathogen and pest attack (Figures 1 and 2). These dynamic modifications of the cell wall enhance mechanical resistance and create biochemical barriers against biotic stressors.

### 3.1 Activation of Monolignol Biosynthesis Upon Pathogen and Pest Attack

Transcriptomic analyses across diverse plant species have consistently demonstrated that biotic stresses induce significant upregulation of phenylpropanoid pathway genes, particularly those encoding enzymes involved in monolignol biosynthesis, ultimately lead to increased lignin accumulation. In Arabidopsis, infection by *Pseudomonas syringae*, *Plasmodiophora brassicae*, or infestation by *Brevicoryne brassicae* triggers substantial transcriptional reprogramming of the phenylpropanoid pathway [52, 53]. Similar responses occur in tomato and grapevine following infection by *Verticillium dahlia* and *Colletotrichum viniferum*, respectively, where the phenylpropanoid pathway exhibits the highest number of differentially expressed genes [54, 55]. This conserved defense response extends to crop species. Rice plants infected with *Xanthomonas oryzae* display pronounced activation of phenylpropanoid metabolism [56]. Tobacco plants infested by the stem borer *Trichobaris mucorea* show upregulated expression of lignin biosynthetic genes, particularly in pith tissues [51]. Wheat challenged with *Puccinia striiformis* and maize inoculated with *Puccinia polysora* and *Fusarium verticillioides* exhibit similar transcriptional induction of lignin‐related genes [57, 58, 59]. Notably, pathogen‐induced activation of these genes not only enhances monolignol production but also promotes accumulation of antimicrobial phenolic compounds. Intriguingly, comparable transcriptional activation occurs in autoimmune and lesion‐mimic mutants even under non‐stress conditions [60, 61, 62], suggesting constitutive activation of lignin‐related pathways in these genetic backgrounds.

In addition to quantitative changes, dynamic alterations in lignin composition are also observed in plants when challenged by various pathogens and pests. Gymnosperms predominantly accumulate H‐lignin under biotic stress, while angiosperms preferentially synthesize S‐lignin [49]. The defensive role of S‐lignin is well documented, as evidenced by its preferential accumulation in infected tissues and the enhanced disease resistance observed in transgenic plants with elevated S‐lignin levels [61, 63, 64, 65, 66, 67]. In contrast, G‐lignin, characterized by more stable biphenyl and carbon‐carbon linkages compared to the ether‐dominated bonds in S‐lignin, demonstrates distinct defensive properties [68, 69]. The interaction between tobacco and *Trichobaris mucorea* reveals additional complexity, showing a decreased S/G ratio in infested stem and increased β‐aryl ether (β–O–4) lignin units in pith tissues, accompanied with accumulation of feruloyltyramine, an atypical lignin monomer with insect‐deterrent properties [51]. These findings collectively demonstrate that biotic stress induces not only quantitative increases in lignin deposition but also qualitative modifications in lignin composition, enabling plants to mount tailored defensive responses through selective monomer incorporation, altered linkage patterns, and the production of specialized defensive compounds against diverse pathogens and pests.

### 3.2 Acceleration of Monolignol Transport Under Biotic Stresses

Both ABC transporters and autophagy are implicated in pathogen‐triggered monolignol transport (Figure 2). For instance, the sinapyl alcohol transporter gene *Lr34* confers durable resistance against *Puccinia triticina* (leaf rust), *Puccinia striiformis* (stripe rust), and *Blumeria graminis* (powdery mildew) in wheat [27, 70]. Wheat lines carrying the *Lr34res* allele exhibit thicker cell wall and higher lignin content compared to the *Lr34sus* allele, while *lr34* mutant lines show hypersensitivity to sinapyl alcohol. Besides, overexpression of *TaCOMT‐3B* gene enhances sinapyl alcohol production and strengthens *Lr34*‐mediated resistance [27]. In Arabidopsis, the plasma membrane‐localized PDR‐type ABC transporter PEN3 accumulates at infection sites to block non‐host fungal penetration [71, 72]. Its substrate, 4‐methoxyindol‐3‐yl methanol, additionally promotes flg22‐induced callose deposition [73]. These findings suggest that its analogous ABC transporters, such as ABCG29, and Lr34, may also be recruited to infection sites for targeted monolignol delivery, thereby enhancing lignification and strengthening disease resistance.

Recent work by Jeon et al. (2023) further demonstrates the potential involvement of autophagy in monolignol transport during pathogen infection. *atg5‐1* and *atg7‐2* mutants exhibit impaired lignin deposition during pathogen response [45]. Live imaging reveals pathogen‐inducible co‐localization of monolignols with autophagic vesicles that traffic along cytoskeletal elements toward the plasma membrane in *Pseudomonas syringae* pv. *tomato* (*Pst*) DC3000‐infected cells, indicating an emergency transport mechanism for rapid cell wall reinforcement [45]. This autophagy‐dependent pathway enables timely lignin barrier formation and helps restrict pathogen spread.

### 3.3 Induction of Monolignol Polymerization Under Biotic Stresses

Monolignol polymerization, catalyzed by laccases (LAC) and peroxidases (PRX), is closely associated with reactive oxygen species (ROS) production during pathogen infection. Pathogen‐induced ROS burst promotes monolignol polymerization, as demonstrated by the strong induction of *GhLAC15* expression in cotton following *Verticillium dahliae* infection, which correlates with enhanced disease resistance [74]. In *Physcomitrella patens, Colletotrichum gloeosporioides* infection upregulates *DIR* expression, enhancing lignin polymerization and cell wall reinforcement [75]. Similarly, *GhDIR1* overexpression in cotton promotes lignification in epidermal and vascular tissues, effectively restricting *Verticillium dahlia* spread [76]. In contrast, *TaDIR‐B1* knockout mutants and VIGS lines in wheat show enhanced lignin accumulation and improved resistance to *Fusarium* crown rot [77]. These data implied that DIR proteins may differentially regulate lignification depending on plant‐pathogen systems.

Casparian strip membrane domain proteins (CASPs) contribute significantly to defense‐related lignification. In Arabidopsis, *CASPL1D1* and *CASPL4D1* are transcriptionally upregulated in response to *Pst* DC3000 and are essential for pathogen‐induced lignin polymerization [69]. Mechanistically, CASPs recruit peroxidases to catalyze monolignol polymerization during Casparian strip formation, utilizing ROS (specifically H_2_O_2_) generated by RBOHF [78]. Given RBOHF‐derived ROS serves as a key immune signaling molecules [79], their involvement in CASPL‐mediated lignification highlights an intricate crosstalk among oxidative signaling, cell wall fortification, and plant immunity.

## 4 Multi‐Layered Regulatory Network Involved in Pathogen‐ and Pest‐Triggered Lignification

Plants possess sophisticated mechanisms to detect pathogen invasion or pest attacks and rapidly initiate protective lignin deposition. Under various biotic stresses, there appear to be several layers of regulation, including transcription factor‐mediated transcriptional control, microRNA‐mediated post‐transcriptional regulation, and protein modification‐dependent post‐translational modifications, resulting in altered lignin accumulation (Figure 2).

### 4.1 Transcriptional Control of Lignin Deposition Under Biotic Stresses

Emerging evidence demonstrates that monolignol biosynthesis in plants is coordinately regulated by the NAC and SG2‐type R2R3‐MYB transcriptional cascade [80, 81]. In Arabidopsis, the SG2‐type R2R3‐MYB transcription factor MYB15 serves as a central regulator of defense‐induced lignification in both PTI and ETI [68, 82]. *MYB15* expression is upregulated by pathogen‐associated molecular patterns (PAMPs), such as flg22 and elf26, and the bacterial effector AvrRpm1. Notably, *MYB15* loss‐of‐function mutants fail to induce lignification in response to these elicitors [68, 82, 83]. Mechanistically, MYB15 directly binds to the promoters of key monolignol biosynthetic genes, including *PAL1*, *C4H*, *HCT*, *CCoAOMT*, *CSE*, and *CAD*, to activate their expression during pathogen attack [68]. Similarly, MYB15 orthologs promote lignification in rice, cotton, apple, and chrysanthemum upon challenges of various pathogens and pests [20, 84, 85, 86, 87, 88]. Notably, MYB15‐mediated defense activation specifically promotes G‐lignin biosynthesis without affecting S‐lignin production (Chezem et al., 2017), suggesting that different monolignols play specialized roles in pathogen‐specific disease resistance.

Beyond MYBs, other transcription factors also contribute to pathogen‐responsive lignification. In Arabidopsis, AtMYC2 orchestrates the shift from chewing pest‐initiated jasmonic acid (JA) signaling into abscisic acid (ABA)‐mediated sustained activation of *RAP2.6*, thereby maintaining monolignol biosynthesis and lignin deposition required for wound healing [25]. In cotton, *Verticillium dahliae* infection induces GhWRKY1, which activates *GhPAL6* and *GhCOMT1* transcription to promote monolignol biosynthesis [65]. In persimmon, DkWRKY and DkWRKY10 mediate *Colletotrichum horii*‐induced lignification through transcriptional regulation of *DkCAD1* [89]. In apple, *Fusarium solani* triggers *MdPRX63*‐mediated monolignol polymerization via *MdWRKY75‐MdERF114* transcriptional cascade and the *MdMYB8‐MdERF114* interaction [90]. Collectively, these studies demonstrate that activation of lignin deposition through transcription factors is an evolutionarily conserved mechanism during plant‐pathogen interactions.

### 4.2 MicroRNA‐Mediated Post‐Transcriptional Regulation of Lignin Deposition Under Biotic Stresses

MicroRNA‐transcription factor modules represent key regulatory mechanisms enabling plants to perceive biotic threats and activate monolignol biosynthesis and polymerization. The induction of *MYB* expression by biotic stress often involves miRNA‐mediated regulation. For example, in rice, infections by *Magnaporthe oryzae*, *Rhizoctonia solani*, and *Xanthomonas oryzae* pv. *oryzae* induce the expression of *OsmiR172a*, which suppresses *OsSNB* and consequently releases *OsMYB30* to activate lignin biosynthesis and confer broad‐spectrum resistance [24]. Similarly, in apple, *Colletotrichum gloeosporioides* infection elevates salicylic acid (SA) levels, alleviating MdmiR7125‐mediated repression of *MdARF1* and subsequently activating MdMYB46‐dependent lignin biosynthesis [20]. In maize, *Fusarium graminearum* and *Pythium inflatum* infection induce the expression of miR319, which in turn targets *ZmMYB74* to promote lignin accumulation and thus enhance disease resistance [31]. The expression of *LAC* gene is also post‐transcriptionally regulated by miR397, whose expression is suppressed upon infection by various pathogens, including *Magnaporthe oryzae* in rice, *Verticillium dahliae* in cotton, *Pythium ultimum* in apple, and *Botrytis cinerea* in *Malus hupehensis* [28, 91, 92, 93, 94]. Through miRNA‐mediated post‐transcriptional regulation, pathogens up‐regulate monolignol biosynthesis and polymerization, ultimately promoting lignin deposition.

### 4.3 Protein Modification‐Dependent Regulation of Lignin Deposition Under Biotic Stresses

Post‐translational modifications (PTMs) represent a fundamental regulatory mechanism that modulates protein stability, activity, localization, and conformation, and they play pivotal roles in plant‐pathogen interactions [95]. The importance of PTMs in fine‐tuning lignin biosynthesis has been highlighted [96]. For example, in Arabidopsis, PAL undergoes multiple PTMs, including phosphorylation by CDPK and ubiquitination mediated by Kelch domain‐containing F‐box (KFB) proteins KFB01, KFB20, KFB39, KFB50, and SAGL1 [97, 98, 99, 100]. Pathogens have evolved strategies to manipulate PTMs in lignin biosynthesis. The vascular bacterial pathogens *Xanthomonas oryzae* pv. *oryzae* and *Xanthomonas campestris* pv. *campestris*, inhibits the phosphorylation of MYB4, a negative regulator of lignin biosynthesis, through interference with MAPK singling cascade. This suppression of MYB4 phosphorylation, promotes lignin accumulation and enhances vascular immunity [101]. Additionally, glycosylation of phenylpropanoid compounds contribute to plant immunity by modulating metabolite stability and activity [102].

Recently, emerging evidence reveals crucial roles for PTMs, such as phosphorylation, ubiquitination, and oxidization, in regulating monolignol biosynthetic enzymes during defense‐induced lignification. In cassava, MeKIN10 phosphorylates MeHB16 dimers to activate monolignol biosynthesis and polymerization during *Xanthomonas axonopodis* pv. *manihotis* infection [26]. In rice, the key lignin biosynthetic enzyme, PAL, undergoes pathogen‐regulated ubiquitination by OsFBK16 and OsPUB41, with *Magnaporthe oryzae* suppressing *OsFBK16* while inducing *OsPUB41* expression [103]. Additionally, *Magnaporthe oryzae*‐ generated H_2_O_2_ oxidizes bHLH25 at M256, stimulating *OsLAC* expression and promoting lignin accumulation [28].

Collectively, these findings demonstrate that plants employ multi‐layered regulatory networks involving transcriptional, post‐transcriptional, and post‐translational mechanisms to precisely modulate lignification in response to diverse biotic stresses. These regulatory processes are intricately linked with the signaling pathways of defense phytohormones, particularly SA, JA, and ABA, which play pivotal roles in fine‐tuning lignification in response to biotic stresses. A key knowledge gap, however, remains in the upstream signaling events that initiate this pathway, particularly regarding how plants perceive biotic stress signals, transduce them, and ultimately activate the expression of lignin‐related genes to induce lignification.

## 5 Alteration of Plant Immunity by Manipulations of Lignin‐Related Genes

Extensive researches have demonstrated that genetic modifications of lignin‐related genes significantly impact plant disease resistance through alterations in lignin content and composition (Table 1).

### 5.1 Alteration of Plant Immunity by Manipulation of Monolignol Biosynthetic Genes

PAL, the entry point of the phenylpropanoid pathway, plays a pivotal role in plant defense. Arabidopsis *pal* mutants, *pal1 pal2* double mutant, and *pal1/2/3/4* quadruple (*palQ*) mutant, show reduced lignin accumulation and increased susceptibility to *Pst* DC3000 [69, 104]. Conversely, overexpression of *PAL* enhances resistance in tobacco against *Phytophthora parasitica*, *Cercospora nicotianae*, and tobacco mosaic virus, and in rice against *Nilaparvata lugens*, *Xanthomonas oryzae pv. oryzae*, and *Magnaporthe oryzae* [21, 105, 106, 107, 108]. Supplementing coniferyl alcohol in the Arabidopsis *palQ* mutant restored lignin deposition and improved disease resistance, highlighting the critical role of lignin in PAL‐dependent disease resistance [69].

HCT catalyzes the conversion of ρ‐coumaroyl CoA to coumaroyl shikimate and caffeoyl shikimate to caffeoyl CoA, pivotal steps for G‐ and S‐lignin biosynthesis. Suppression of *HCT* in *Medicago sativa* reduces lignin levels but enhances resistance to *Colletotrichum trifolli* [109], suggesting negative regulation of plant immunity. Additionally, two maize HCT homologs, HCT1806 and HCT4918, physically interact with the R protein Rp1‐D21, leading to the suppression of Rp1‐D21‐mediated cell death [110, 111], implying that HCT have lignin‐independent function in plant immunity.

CCR catalyzes the conversion of feruloyl‐CoA to coniferaldehyde. Arabidopsis *ccr1‐3* mutant shows ectopic defense gene expression [112], while contrasting roles are observed for *ccr1‐6* (increased susceptibility to *Pst* DC3000) and *Brassica napus CCR2*‐overexpressing lines (enhanced resistance to *Sclerotinia sclerotiorum*) [68, 113]. Although both *ccr1‐3* and *ccr1‐6* have similar lignin levels, *ccr1‐3* exhibits more severe growth defects and greater cellulose reduction [114], suggesting that defense responses are triggered by substantial cell wall disruption.

4CL catalyzes the formation of hydroxycinnamoyl‐CoA esters, essential precursors for monolignol biosynthesis. Genetic evidence demonstrates the positive role of 4CL in plant immunity. The loss‐of‐function mutation of *Gh4CL3* in cotton and RNA interference (RNAi) of *Cm4CL2* in *Cucumis melo* cause reduced lignin content and increased susceptibility to *Verticillium dahliae* and aphid *Macrosiphoniella sanborni*, respectively, while their overexpressing lines have opposite effects [84, 115]. However, a contrasting mechanism is observed in cotton, where *Gh4CL30* silencing reduces lignin content but enhances *Verticillium* resistance due to the accumulation of antimicrobial phenolic compounds caffeic acid and ferulic acid [116]. These findings collectively demonstrate that 4CL‐mediated defense responses involve both lignin‐dependent and lignin‐independent mechanisms, depending on the specific plant‐pathogen interaction and the metabolic interference.

Two key *O*‐methyltransferases (OMTs), CCoAOMT and COMT, mediate phenyl ring methylation in G‐ and S‐lignin biosynthesis. Arabidopsis *ccoaomt1‐3* and *ccoaomt1‐5* mutants exhibit increased susceptibility to *Pst* DC3000 and *Hyaloperonospora arabidopsidis*, while overexpression of *ZmCCoAOMT2* enhances resistance to southern leaf blight in maize, demonstrating *CCoAOMT’*s positive role in defense [117]. However, the defense role of *COMT* shows pathogen‐specificity. Suppression of *COMT* in tobacco reduces S‐lignin content but improves pathogen resistance, potentially by limiting phenolic virulence inducer production [118]. Arabidopsis and *Sorghum bicolor comt* mutants also display enhanced resistance to *Hyaloperonospora arabidopsidis* and *Fusarium* spp [119, 120]. Paradoxically, the knock‐out mutation or gene silencing of *COMT* homolog genes cause increased susceptibility to multiple diseases in Arabidopsis, maize, and wheat [27, 121, 122]. COMT's complex immune functions may stem from its interaction with the Rp1‐D21 resistance protein in maize, where it cooperates with HCT to regulate hypersensitive responses [110]. These contradictory observations highlight the context‐dependent nature of COMT‐mediated immunity, requiring further mechanistic investigation.

F5H catalyzes the hydroxylation of the monolignol precursors’ aromatic ring, shifting biosynthesis from G‐lignin to S‐lignin production. Arabidopsis *f5h1* mutant exhibits reduced S‐lignin and sinapate esters, resulting in elevated G/S ratio and increased susceptibility to fungal pathogens *Sclerotinia sclerotiorum* and *Verticillium longisporum* [123, 124], while overexpression of *F5H* exhibits enhanced defense against *Pst* DC3000 but greater vulnerability to *Myzus persicae* [63].

As the final enzymatic step in monolignol biosynthesis, CAD catalyzes the reduction of cinnamyl aldehydes to their corresponding alcohols. Silencing the *CAD* gene in flax and *cad* mutants in maize and rice (*zmcad*, *oscad8b*, and *oscad2*) exhibit decreased lignin accumulation and increased susceptibility to *Fusarium oxysporum, Rhizoctonia solani*, and *Magnaporthe oryzae*, respectively [121, 122, 125, 126, 127]. Conversely, overexpression of *CAD* leads to increased lignification and enhanced resistance against *Bsorokiniana sorokiniana* and *Fusarium pseudograminearum* in wheat, *Colletotrichum horii* in persimmon, and *Botrytis cinerea* in rose [18, 89, 128]. These studies collectively support a broadly positive role for CAD in plant immunity. However, the sorghum *bmr6*/*cad* mutant represents a notable exception, showing suppressed *Fusarium* spp. growth, potentially due to the accumulation of antimicrobial intermediates or constitutive activation of defense pathway [119].

### 5.2 Alteration of Plant Immunity by Manipulation of Genes Involved in Monolignol Transport and Polymerization

Recent genetic evidence demonstrates that mutations in the sinapyl alcohol transporter gene *Lr34* (*m19* and *m21*) reduce both lignin accumulation and resistance to multiple fungal pathogens [27], underscoring the importance of monolignol transport in plant defense. Key enzymes involved in lignin polymerization, including LAC, PRX, CASPL, and DIR, exert significant influence on disease resistance through distinct mechanisms. In rice and kiwifruit, overexpression of *OsLAC* and *AcLAC35* cause increased lignin accumulation and disease resistance [28, 129]. *GhLAC15* is induced by *Verticillium dahlia* in cotton, and overexpression of *GhLAC15* in Arabidopsis also confers increased resistance to *Verticillium* wilt, concomitant with elevated G‐lignin and G/S ratio [74]. Conversely, suppression of *LAC*s compromises lignin deposition and disease resistance in rice and cotton [28, 74, 91].

Similar regulatory patterns are extended to other polymerization components. The *MdPRX63* overexpression lines show improved *Fusarium solani* resistance in apple [130], while Arabidopsis *CASPL*‐deficient lines, *caspl4d1‐1*, *caspl4d1‐2*, and *amiCASPL1D* exhibit reduced lignin and increased *Pst* DC3000 susceptibility [82]. Manipulation of DIR proteins also affects disease resistance, as overexpression of *GhDIR1* in cotton delays *Verticillium dahliae* invasion through enhanced lignification [76], and wheat *TaDIR‐B1* deficiency increases both lignin content and *Fusarium* crown rot resistance [77]. Collectively, these findings demonstrate that genetic manipulation of monolignol transport and polymerization components can substantially alter plant immunity through modulation of lignin deposition patterns.

### 5.3 Alteration of Plant Immunity by Manipulation of Regulatory Genes in Lignin Pathways

MYB transcription factors serve as master regulators of both lignin biosynthesis and plant immunity, exhibiting complex functional diversity across species. Some MYB transcription factors, such as *AtMYB15*, *CmMYB15*, *GhODO1*, *GhMYB3D5*, *MdMYB46*, and *OsMYB30*, act positive regulator of lignin biosynthesis. Mutations or suppression of these *MYB* genes consistently reduce lignin accumulation and compromise resistance in various biotic stress, such as *Pst* DC3000 in Arabidopsis, aphid *Macrosiphoniella sanborni* in chrysanthemum, *Verticillium dahliae* in Cotton, *Colletotrichum gloeosporioides* in apple, and multiple pathogens in rice [20, 24, 60, 82, 86, 88]. In contrast, overexpression of some of these genes, including *CmMYB15*, *GhMYB3D5*, *MdMYB46*, and *OsMYB30*, enhances lignin deposition and strengthens plant resistance to corresponding pathogens or pests [20, 24, 60, 86]. Several other MYB proteins, such as AtMYB4, OsMYB102, OsMYB108, ZmMYB74, and ZmMYB31, act as negative regulators of lignin biosynthesis [31, 101, 131]. Overexpression of these genes cause reduced lignin accumulation and disease resistance, whereas suppression of them cause increased lignin accumulation and enhanced disease resistance [31, 101, 131]. Notably, *GhMYB4* represents an exception: although its overexpression leads to reduced lignin accumulation, it enhances resistance to *Verticillium dahlia*, likely due to activation of JA‐mediated defense [87].

Beyond MYBs, WRKY transcription factors also significantly influence lignin‐mediated defense. As described above, lignin deposition is modulated by miRNAs and PTMs. Therefore, perturbing these regulatory components results in altered lignin accumulation and corresponding shifts in pathogen or pest resistance. For instance, overexpression of *miR172a* elevates lignin content and enhances disease resistance in rice [24]. Conversely, overexpression of *miR397* reduces lignin accumulation and compromises disease resistance in rice, cotton, *Malus hupehensis*, and *Camellia sinensis*, while its suppression has opposite effects [28, 91, 92, 94]. Genetic studies in Arabidopsis and rice demonstrate that a loss‐of‐function mutant of *MKP1* reduces disease resistance, whereas overexpression of *MKP1* or knockout of *MPK3*/*6* enhances pathogen defense [101]. Besides, ZmPTI1c interacts with and phosphorylates ZmMYB31, a negative regulator of lignin biosynthesis [131]. Consequently, the *zmpti1c* mutant exhibits enhanced resistance to *Fusarium graminearum*, which coincides with increased lignin accumulation [131]. In addition, the tetratricopeptide repeats (TPRs)‐containing protein BSR‐K1 regulates *OsPAL1* mRNA turnover in rice, with *bsr‐k1* mutant showing enhanced resistance to *Magnaporthe oryzae* and *Xanthomonas oryzae* pv. *oryzae* through increased lignification [108]. The expression of *LAC* is also up‐regulated in the loss‐of‐function mutant of *ZmSKI3*, a close homolog of *BSR‐K1*, which displays enhanced disease resistance to *Curvularia lunata* in maize [132]. Furthermore, the F‐box protein OsFBK16 and U‐box protein OsPUB41 mediate OsPAL1 degradation, and mutation of either gene improves resistance to rice blast and brown planthopper [19, 103, 107].

### 5.4 Genetic Manipulation of Lignin‐Related Genes Reveals Lignin's Multifaceted Roles in Plant Immunity and Growth

Genetic studies firmly establish that enhancing the lignin pathway generally provides broad‐spectrum resistance against a wide range of biotrophic and necrotrophic pathogens and pests (Table 1). The recurrent pattern across species is that genetic interventions that bolster lignification frequently strengthen physical and chemical barriers. However, the numerous exceptions and context‐dependent outcomes reveal that lignin's role is deeply embedded within a complex metabolic network. Extensive research demonstrates lignin's dual role in plant immunity, with predominantly positive effects in pathogen resistance but context‐dependent negative impacts when lignification interferes with cell wall dynamics or phytoalexin biosynthesis. The mechanisms underlying these paradoxes can be attributed to the redirection of the phenylpropanoid pathway or immune activation. For example, reduced HCT activity in alfalfa leads to the accumulation of flavonoids that act as potent antimicrobial compounds and trigger constitutive activation of defense responses by impaired secondary cell wall integrity [109]. Similarly, silencing *Gh4CL30* in cotton reduces lignin content but increases ferulic acid and caffeic acid accumulation so as to enhance resistance to *Verticillium dahliae* [116]. In addition, perturbations of lignin can redirect carbon flux, alter signal dynamics, and unveil trade‐offs between growth and defense. For instance, lignin‐deficient mutants, such as the Arabidopsis *c4h/ref3* mutant, *bpb1* mutant, and rice *Os4CL3*‐suppressing line, exhibit pronounced growth and developmental defects [133, 134, 135], and over‐immunity mediated abnormal deposition of lignin arrests the normal enlargement of the root tubers of *Rehmannia glutinosa* [136].

## 6 Physiological and Biochemical Basis of Lignin‐Associated Plant Immunity

**FIGURE 3:**
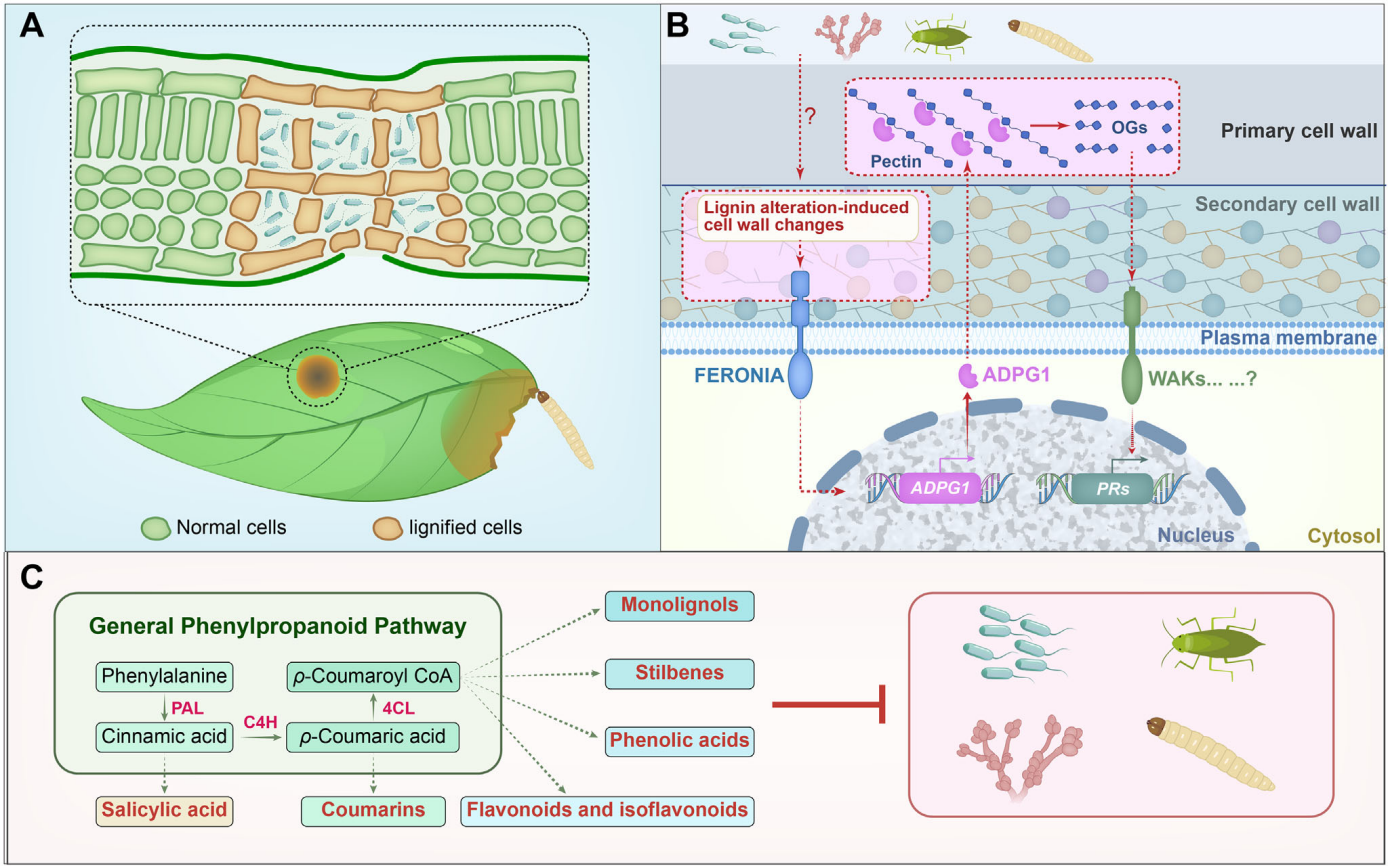
Molecular Mechanism of Lignin‐mediated Plant Immunity. Lignin contributes to plant defense through three primary molecular mechanisms: (A) As an inducible physical barrier, lignin polymerization at infection sites creates a hydrophobic matrix that physically restricts pathogen colonization and spread while simultaneously deterring pest feeding through enhanced mechanical resistance; (B) The phenylpropanoid pathway enables simultaneous production of monolignols, salicylic acid, and antimicrobial phytoalexins, including stilbenes, phenolic acids, flavonoids, and isoflavonoids. (C) Lignin biosynthetic gene mutations‐ or pathogen‐induced lignin alteration trigger cell wall integrity surveillance systems and activate the expression of *ADPG1* through the receptor‐like kinase FERONIA. Then ADPG1 degrades pectin to directly or indirectly release a range of oligosaccharides (OGs) that activate pattern‐triggered immunity and downstream defense signaling cascades. These interconnected mechanisms collectively establish a multi‐layered defense system where lignin's structural role synergizes with its capacity to initiate immune signaling and support chemical defense compound biosynthesis.

The physiological and biochemical mechanisms underlying lignin‐mediated immunity can be categorized into three primary modes (Figure 3): **(I) Physical barrier**: Biotic stress‐induced lignin deposition creates a structural barrier that physically impedes pathogen penetration and spread. Enhanced cell wall rigidity and rapid lignin deposition at wound‐sites also deters pest feeding. **(II) Antimicrobial Compounds**: The phenylpropanoid pathway branches to produce both lignin polymers and antimicrobial phytoalexins. Metabolic flux partitioning between these branches influences the balance between structural and chemical defenses. **(III) Immune signaling molecule**: Lignin‐associated cell wall remodeling can release endogenous elicitors that activate pattern‐triggered immunity, amplifying downstream defense signaling cascades.

### 6.1 Defense Lignin as a Physical Barrier Against Pathogens and Pests

Lignin is a well‐established structural component in plant cell walls. In plant defense, it functions as both a constitutive and inducible physical barrier against pathogen invasion and pest herbivory [12, 137]. While lignin universally enhances cell wall mechanical strength, emerging evidence reveals distinct properties between developmental lignin and stress‐induced “defense” lignin that exhibits structural variations depending on stress type (biotic or abiotic) and plant species involved [49]. Cutting‐edge investigations have substantially advanced our understanding of defense lignin's barrier functions, particularly through molecular characterization of its dynamic formation and spatial deposition in response to various biotic threats [45, 51, 69].

By analyzing lignin deposition patterns in Arabidopsis seedlings and mature plants challenged with various *Pst* strains, including virulent (*Pst* DC3000) and avirulent stains (*AvrRpm1* and *AvrRpt2*, ETI‐activating stains; *hrcC^−^*, PTI‐activating stains), Lee et al. revealed that immune‐activating strains (both ETI‐ and PTI) induced substantially greater lignin accumulation than the virulent strain, particularly at infection sites [69]. Advanced cytological imaging using specialized fluorescent lignin probes further demonstrated precise spatial patterning of lignin deposition surrounding bacterial invasion sites, forming a restrictive “cage” structure that effectively limited pathogen mobility (Figure 3A). Molecular characterization identified CASPL1D1 and CASPL4D1 as key mediators of this pathogen‐induced lignification process, which work in concert with an autophagy‐dependent monolignol transport system that rapidly deliver phenolic precursors to the infection sites, reinforcing cell walls during pathogen attack [45, 69]. This coordinated mechanism establishes a specialized mechanical barrier analogous to the Casparian strip, with CASPL proteins serving as evolutionarily conserved components of lignin‐based extracellular compartmentalization. Similar lignified boundary structures are observed in diverse plant tissues, including root Casparian strips and cucumber glandular trichome neck strips [138, 139].

Transcriptomic profiling of insect‐damaged tobacco revealed pith‐specific induction of lignin biosynthetic genes, resulting in localized accumulation of lignin polymers containing feruloyltyramine as a specialized structural component that functions as a stem‐specific defensive barrier against wood‐boring pests [51]. Inducible lignification process is coordinately regulated by JA and ethylene signaling cascades. Parallel investigations in maize demonstrate that *Spodoptera frugiperda* infestation triggers emission of phytol volatile compounds, which act as interplant warning signals to activate preemptive defense responses in neighboring plants through ethylene‐mediated lignin deposition [140]. Furthermore, mechanical wounding from pest feeding initiates a biphasic hormonal response, an initial JA burst followed by sustained ABA signaling, that orchestrates prolonged lignin biosynthesis for tissue reinforcement and wound repair [25]. Collectively, these findings establish lignification as a dynamically regulated defense mechanism wherein plants precisely tailor their cell wall reinforcement strategies in response to distinct biotic threats, employing both localized and systemic signaling pathways to optimize physical barrier formation against diverse pathogens and pests.

### 6.2 Antimicrobial Intermediate Compounds in Lignin Biosynthesis

The phenylpropanoid pathway producing both structural lignin and various antimicrobial phytoalexins, including coumarins, phenolic acids, stilbenes, flavonoids, and isoflavonoids etc., thereby playing dual roles in plant defense [141, 142]. (Figure 3B) In addition, this pathway produces SA, a crucial phytohormone in plant defense [143, 144, 145]. Metabolic flux redirection in lignin‐deficient plants often enhances phytoalexin production, as demonstrated in cotton, where *Gh4CL30* silencing reduces lignification but increases accumulation of antimicrobial caffeic acid and ferulic acid, conferring resistance to *Verticillium dahliae* [116]. Remarkably, certain defense compounds can be incorporated into lignin polymers, exemplified by the detection of feruloyltyramine in lignin of tobacco stem pith following pest attack [51]. These findings illustrate the metabolic flexibility of the phenylpropanoid pathway in coordinating both chemical and structural defense strategies.

### 6.3 Lignin‐Mediated Cell Wall Remodeling Triggers Plant Immunity

Lignin contributes to plant immunity through dynamic cell wall remodeling that activates defense signaling pathways. In *HCT*‐suppressing alfalfa, reduced lignification correlates with elevated SA and JA levels and upregulation of stress‐responsive genes [109]. The molecular mechanism is further clarified through the lignin‐deficient mutant *ccr1‐3* [112, 146]. This immune activation involves a FERONIA‐mediated pathway where pectin degradation by *ARABIDOPSIS DEHISCENCE ZONE POLYGALACTURONASE 1* (*ADPG1*) releases oligogalacturonides (e.g., GalA3) that potentially initiate defense responses through wall‐associated kinases (WAKs), (Figure 3C) though recent evidence questions WAKLs' essential role in oligosaccharide‐induced immunity [147]. FERONIA additionally regulates resistance through RD26 phosphorylation, which modulates lignin biosynthesis during *Ralstonia solanacearum* infection [23]. Other cell wall integrity sensors like THESEUS1 (THE1) and MALE DISCOVERER 1‐INTERACTING RECEPTOR‐LIKE KINASE 2 (MIK2) also participate in this complex signaling network [148]. Key unanswered questions remain regarding the primary receptors that mediate lignin‐deficiency‐induced immunity, as well as the potential differences in immune activation resulting from genetic mutation‐induced lignin alterations versus pathogen‐triggered lignin remodeling.

## 7 Improvement of Multiple Disease Resistance by Manipulating Lignin‐Related Genes in Major Crops

Both forward and reverse genetics have established the potential for leveraging lignin to enhance crop disease resistance. Hence, dissecting existing successful cases and conceptualizing novel technical strategies is pivotal for guiding subsequent research.

### 7.1 Successful Cases of Genetic Modification of Lignin Genes for Crop Improvements

**FIGURE 4:**
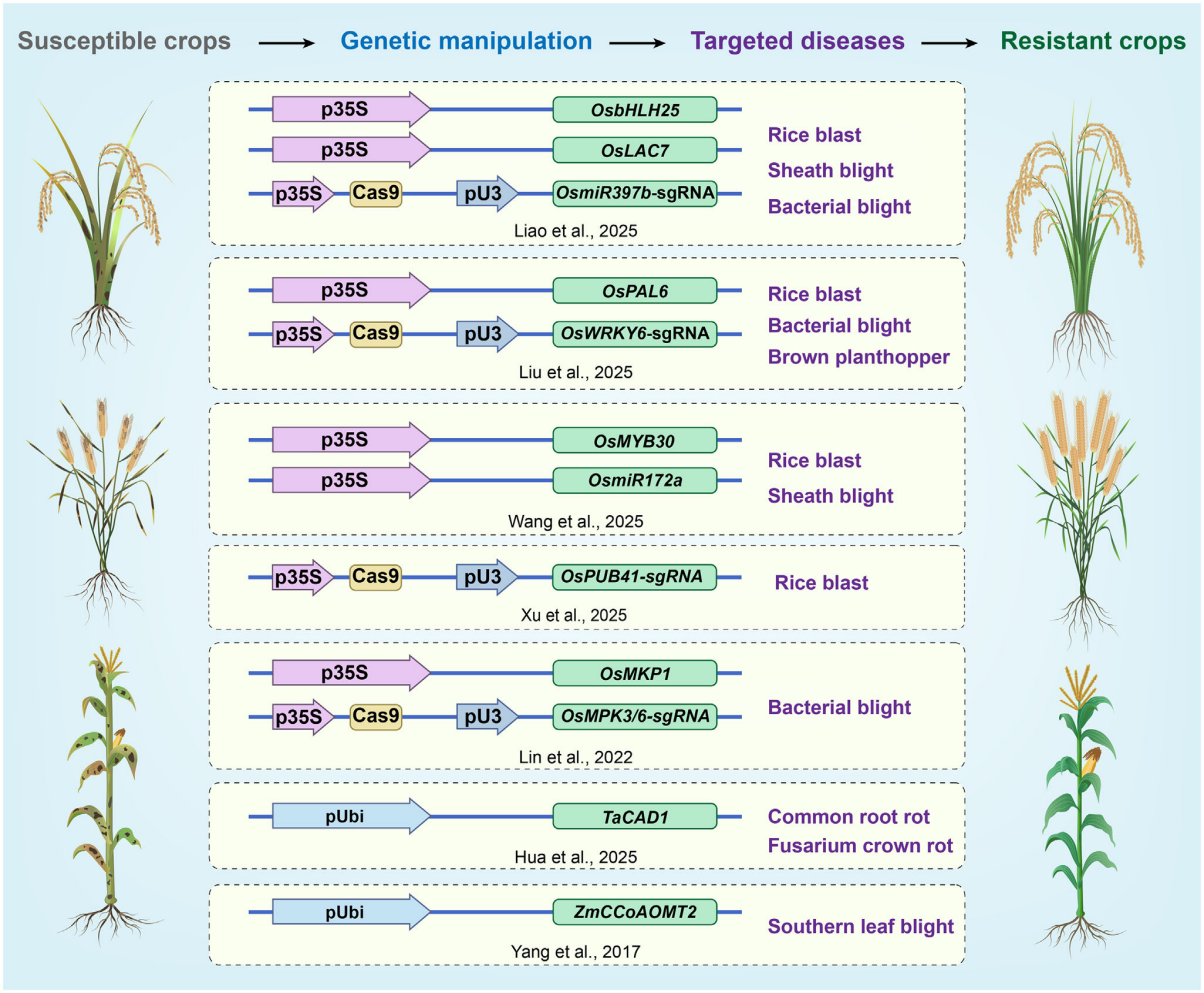
Improved Disease resistance in Crops Through Genetic Manipulation of Lignin‐related Genes. Shown are paradigms of transgenic plants genetically manipulating the lignin‐related genes in rice, wheat, and maize, respectively. p35S, 35S promoter, pUbi, Ubiquitin promoter, pU3, U3 promoter.

Genetic modification of the lignin biosynthetic pathway demonstrates significant potential for enhancing crop disease and pest resistance, as evidenced by successful applications in rice, wheat, and maize (Figure 4). In rice, targeted manipulation of lignin‐related genes by overexpression (*OsbHLH25*, *OsLAC7*, *OsPAL6*, *OsMYB30*, and *OsmiR172a*) or CRISPR/Cas9‐mediated gene editing (*OsmiR397b, OsPUB41*, and *OsWRKY6*) significantly improves resistance to major pathogens and pests, including *Magnaporthe oryzae* (rice blast), *Rhizoctonia solani* (sheath blight), *Xanthomonas oryzae* pv. *oryzae* (bacterial blight), and *Nilaparvata lugens* (brown planthopper), with concurrent increases in lignification [21, 24, 28, 103]. Notably, overexpression of *OsMKP1* and knockout *OsMPK3*/6 improve resistance to the rice vascular disease bacterial blight [21]. In wheat, overexpression of *TaCAD1* confers enhanced resistance against *Bipolaris sorokiniana* (common root rot) and *Fusarium pseudograminearum* (*Fusarium* crown rot) [18]. In maize, overexpression of *ZmCCoAOMT2* confers improved field resistance to *Bipolaris maydis* (southern leaf blight) [117]. These successes highlight lignin pathway engineering as a highly promising strategy for improving broad‐spectrum disease resistance in staple crops.

### 7.2 Natural Allelic Variations of Lignin‐related Genes: Balancing Disease Resistance and Agronomic Traits

Natural allelic variations in lignin‐related genes provide a key genetic resource for enhancing disease resistance while preserving favorable agronomic traits, in contrast to the detrimental effects of artificial genetic modification. In rice, the *bsr‐k1* allele exhibits broad‐spectrum resistance to *Magnaporthe oryzae* and *Xanthomonas oryzae* pv. *oryzae* without compromising yield, through modulation of *OsPAL* transcript stability [108]. Notably, *OsPAL* genes (*OsPAL1‐ OsPAL7*) colocalize with quantitative trait loci (QTL) for multiple disease resistance [149, 150]. Similarly, a natural allele of *OsTB1* enhances both culm strength and blast resistance via promoting lignin biosynthesis [30]. In maize, natural variation at *ZmCCoAOMT2*, located within the qMdr9.02 QTL, contributes to resistance against southern leaf blight, gray leaf spot, and northern leaf blight [117]. In wheat, allelic variation in *Lr34*, encoding a functional sinapyl alcohol transporter, confers multi‐pathogen resistance against *Puccinia triticina*, *Puccinia. striiformis*, and *Blumeria graminis* [27, 70]. These natural variants of lignin pathway genes represent prime targets for precision breeding of broad‐spectrum disease‐resistant crop varieties with minimal yield penalties. While genome‐wide association studies (GWAS) have supported the identification of such nature variants and become a widely accepted strategy for decoding genotype‐phenotype associations [151, 152, 153, 154]. A GWAS of lignin content in 206 globally representative sorghum accessions identified 9 significant QTLs and 184 candidate genes associated with lignification [155]. Similarly, integrated analysis of 9 RIL populations revealed colocalization of key monolignol biosynthetic genes and their regulatory *MYB*/*NAC* transcription factors with lignin‐related QTLs in maize [156]. Several variations of lignin‐related genes associated with disease resistance have also been identified by GWAS. For example, natural variation of *ZmFBL41* is found significantly associated with banded leaf and sheath blight resistance by GWAS with 318 inbred lines [126]. In wheat, allelic variations of *TaDIR‐B1* are found associated with *Fusarium* crown rot resistance by GWAS with 435 introgression lines [77], paralleled by the structural variations of *DIR* gene mediating *Fusarium* wilt disease resistance in *Sesamum* species [22]. The identification of the allelic variations of lignin and disease resistance‐related genes facilitate targeted genetic improvement of lignocellulosic traits and disease resistance in crops.

### 7.3 Biotechnological Strategies of Lignin Engineering for Crop Improvement

**FIGURE 5:**
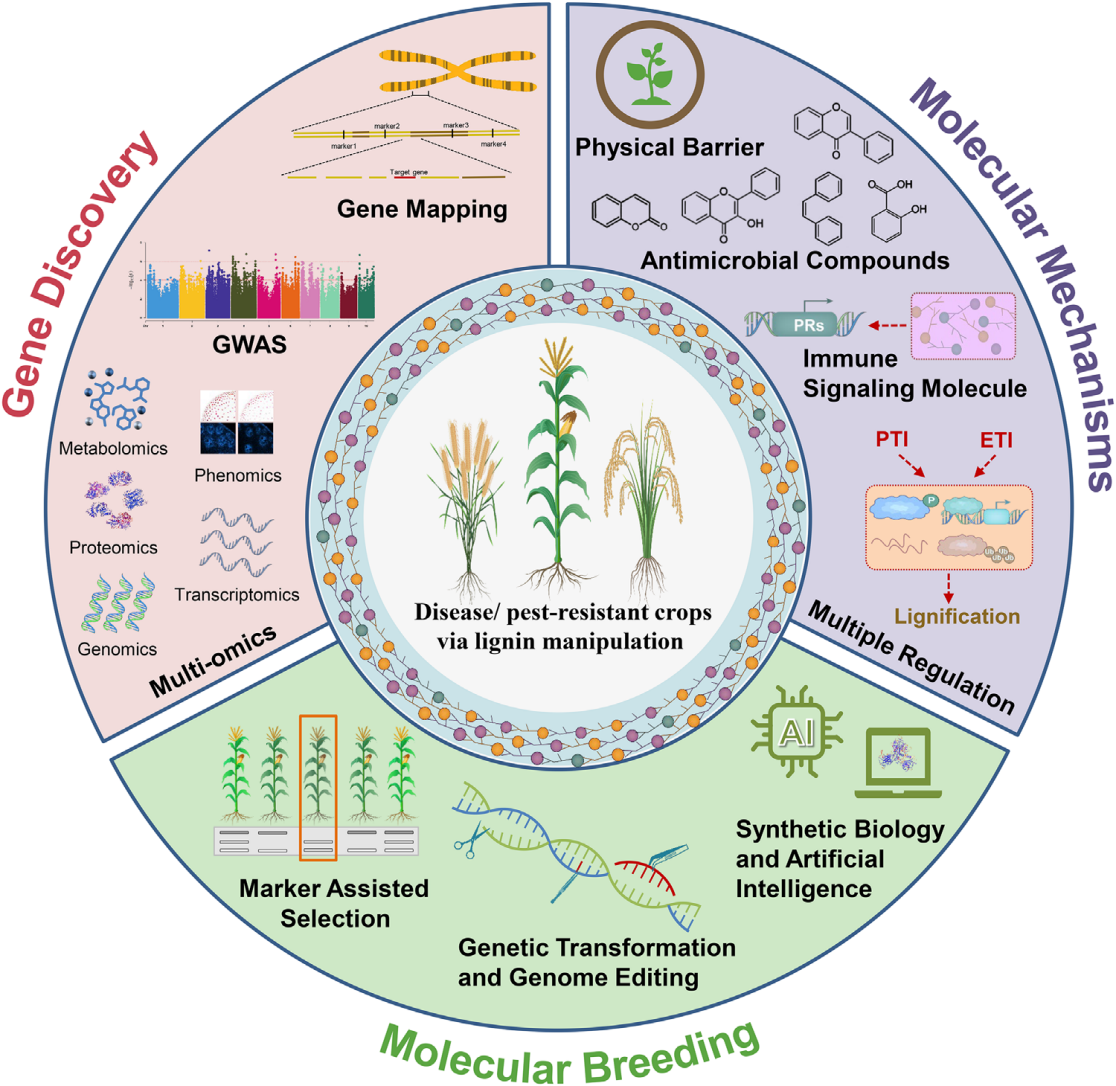
Biotechnological Strategies for Breeding Disease‐ and Pest‐Resistant Crops through Lignin Pathway Engineering.

Manipulation of lignin content and composition has emerged as a promising approach for developing disease‐resistant crop varieties, given the strong correlation between enhanced lignification and improved pathogen resistance. Modern biotechnological tools, including marker‐assisted selection, gene editing, genetic transformation, genomic selection, protein design, and synthetic biology offer unprecedented precision for modifying lignin‐related genes and proteins (Figure 5). Previous synthetic biology efforts focused on lignin valorization and biomass processing has provided valuable foundational knowledge for these applications [157, 158, 159]. For instance, structure‐guided iterative saturation mutagenesis was used to engineer novel OMT variants capable of catalyzing 4‐*O*‐methylation of monolignols. When expressed in aspen and rice, these engineered OMTs altered lignin structure, resulting in enhanced sugar release and improved ethanol yield from biomass [160, 161]. Similarly, reconstructing the lignin biosynthetic pathway can enhance lignification, thereby improving plant resistance to pathogens and pests. This engineering strategy is not limited to modifying monolignol biosynthetic enzymes. Synthetic biology enables the design of chimeric proteins that specifically bind to both monolignols and cell wall polymers such as cellulose [162]. Such proteins can guide monolignols to undergo ordered polymerization at predetermined sites, forming a denser and more stable lignin structure that confers resistance against pathogen and pest attacks. Recently, rapidly emerging artificial intelligence (AI) technologies have shown great potential to facilitate gene editing and crop design [163, 164]. Integrating AI with the expanding dataset on the resistance phenotypes of lignin‐modified plants (Table 1) will further accelerate the development of crops with improved resilience against pathogens and pests through precision lignin engineering. In the future, the breeding practices will be revolutionized by the integration of high‐throughput phenotyping, advanced genomics, and genome editing alongside machine learning and algorithm‐driven analytics [165, 166].

It is important to recognize that lignin modification can impose trade‐offs on plant growth and immunity, as evidenced by developmental abnormalities in lignin‐deficient mutants like the Arabidopsis *c4h*/*ref3* mutant and rice *Os4CL3*‐suppressing line [133, 134]. Previous studies indicate that growth defects in lignin‐reduced plants can be mitigated through modulation of SA signaling or the Mediator complex [167, 168], suggesting viable strategies to avoid growth penalty. Optimal approaches may involve employing pathogen‐inducible or tissue‐specific promoters to precisely control lignin biosynthetic gene expression, or utilizing CRISPR/Cas9 for targeted knockout/knock‐in of lignin‐related genes to develop varieties with coordinated improvement of disease resistance and other agronomic traits.

## 8 Conclusion and Perspectives

Cell wall lignification represents a sophisticated defense mechanism involving coordinated monolignol biosynthesis, transport, and polymerization processes that are activated in response to biotic stresses (Figures 1 and 2). As a major downstream executor of plant immunity, lignin deposition enhances plant resistance through three primary mechanisms (Figure 3): (1) Formation of structural barriers in secondary cell walls that physically impede pathogen invasion and pest feeding; (2) Production of antimicrobial phenolic intermediates that function as phytoalexins; and (3) Activation of immune responses through cell wall Damage‐Associated Molecular Patterns (DAMPs) like GalA3. While extensive evidence supports lignin's positive role in defense, some lignin‐deficient plants exhibit enhanced resistance, likely due to compensatory activation of immune pathways or elevated phytoalexin accumulation rather than lignin deficiency itself. Importantly, not all lignin‐reduced genotypes exhibit immune activation, and it remains unclear whether the pathogens or pests‐induced disturbance of lignification similarly triggers defense responses to enhance plant immunity. Therefore, lignin by itself generally plays a positive rather than a negative role in defense responses against various diseases and pests, serving as a dynamic fortress in the front line of plant immunity. Meanwhile, lignin has garnered considerable interest for its application in plant resistance, notably for its outstanding performance in providing broad‐spectrum resistance (Table 1).

Despite significant progress, our mechanistic understanding of lignin‐mediated plant immunity is limited. Several key knowledge gaps must be addressed. Especially critical knowledge gaps remain in understanding the complete signaling cascades linking biotic stress perception to lignification. (1) Emerging modules like MKP‐MAPK‐MYB4, FERONIA‐RD16, and KIN10‐HB16 highlight the importance of phosphorylation networks in lignin‐mediated defense [24, 26, 69, 101], but upstream receptors and potential convergence points of pathogen/pest‐induced lignification require clarification. It also remains to be investigated if PTI and ETI mediate lignification independently or converge on one shared pathway. (2) While enhanced lignin accumulation confers broad‐spectrum resistance against both necrotrophic and biotrophic pathogens (Table 1), it is unclear whether a shared molecular mechanism underlies this broad‐spectrum defense and how it operates against such divergent pathogen types. (3) It is necessary to elucidate how different lignin constituents (e.g., S and G units) contribute to structural configurations under specific biotic stress, thereby facilitating genetic engineering of lignin composition and structure to improve resistance. Toward those concerns, additional components and pathways involved in pathogen‐ and pest‐activated lignification await in‐depth exploration.

With climate change intensifying disease pressures, lignin manipulation through marker‐assisted selection and precision gene editing offers a promising strategy for developing high‐yielding, resistant crops. Successful cases in rice, wheat, and maize demonstrate lignin's potential as an engineering target toward broad‐spectrum biotic stress resistance with minimal yield penalties [27, 108, 117] (Figure 4). Consequently, manipulating lignin biosynthesis in crops via marker‐assisted selection, genome editing, and AI‐driven biotechnology offers an effective strategy to coordinately improve crop disease/pest resistance and yield (Figure 5). Based on previous research, gene redundancy is a common phenomenon in gene families involved in lignin biosynthesis and regulation, especially in crops like maize, rice, and wheat [169, 170, 171]. Gene redundancy enhances the stability and robustness of the lignin biosynthesis system, ensuring that plants can maintain normal lignin accumulation and defense capabilities under environmental fluctuations or mild gene mutations. However, it poses challenges to crop engineering, as single‐gene modification fails to induce significant changes in lignin content or composition. The improvement of crop resistance through lignin modification may require the manipulation of multiple redundant regulatory genes simultaneously or the identification of the key family members.

In the secondary cell walls, lignin cross‐links with cellulose and hemicellulose to enhance cell wall rigidity, and the roles of these two components in plant immunity have also been studied [172]. There is still no direct evidence that lignin or its derived oligomers can trigger immunity as DAMPs. Thus, the activation of defense in a lignin‐deficient mutant is considered to be due to the impairment of cell wall integrity, including the structure of cellulose and hemicellulose. Recently, a xylan xylosyltransferase gene, *ZmXYXT2*, has been identified in maize that can enhance resistance to *Fusarium verticillioides* through the modulation of hemicellulose biosynthesis [173]. In the overexpression of *ZmXYXT2* lines, not only hemicellulose, but also cellulose and lignin contents are increased [173]. Therefore, lignification, cellulose synthesis, and hemicellulose synthesis are tightly coordinated, and their interactions are crucial for maintaining cell wall integrity and enhancing plant defense. This coordinated regulatory network should be taken into consideration for the genetic improvement of crop disease/pest resistance and yield in the future.

Future research should focus on: (1) Elucidating the complete signaling networks that induce defensive lignification; (2) Optimizing spatial‐temporal control of defense lignin deposition; and (3) Developing integrated breeding and engineering strategies that maximize resistance while maintaining agronomic performance. Leveraging lignin for crop improvement requires a systems‐level understanding that considers the pathway not as a simple linear contributor to barrier formation, but as a dynamic and integrated node within the plant's overall defense economy. Overall, we contend that targeted metabolic engineering of the lignin pathway, informed by mechanistic insights into lignin's immune functions, is a long‐term yet high‐reward strategy to concurrently enhance crop resistance and yield.

## Author Contributions

M.G. conceived the topic. Y.Y. and M.G. wrote the major manuscript and prepared the figures and tables. C.‐J. L, J. L, L. X, H. T, and K.W. reviewed and revised the manuscript. All authors read and approved the final manuscript.

## Funding

This work was supported by the National Natural Science Foundation of China (U24A20395, 32372208 and U2004207 to MG, 32572408 to YY), Rolling Support of Fund for Distinguished Young Scholars in Henan (252300421235 to MG), the Natural Science Foundation of Henan Province (252300423062 to YY), the Henan Province Tackling Key Problems in Science and Technology (252102110257 and 262102110261 to JL, 242103810021 to LX) and U.S. Department of Energy, Office of Science, Office of Basic Energy Sciences (DE‐SC0012704 to CL, through the Physical Biosciences program of the Chemical Sciences, Geosciences and Biosciences Division).

## Conflicts of Interest

The authors declare no conflicts of interest.

## Data Availability

Data sharing not applicable to this article as no datasets were generated or analyzed during the current study.
